# All-optical superconducting qubit readout

**DOI:** 10.1038/s41567-024-02741-4

**Published:** 2025-02-11

**Authors:** Georg Arnold, Thomas Werner, Rishabh Sahu, Lucky N. Kapoor, Liu Qiu, Johannes M. Fink

**Affiliations:** 1 Institute of Science and Technology Austria, Klosterneuburg, Austria; 2 Present Address: QphoX B.V., Delft, the Netherlands; 3 Present Address: School of Physics, Zhejiang University, Hangzhou, China

**Keywords:** Qubits, Applied optics

## Abstract

The rapid development of superconducting quantum hardware is expected to run into substantial restrictions on scalability because error correction in a cryogenic environment has stringent input–output requirements. Classical data centres rely on fibre-optic interconnects to remove similar networking bottlenecks. In the same spirit, ultracold electro-optic links have been proposed and used to generate qubit control signals, or to replace cryogenic readout electronics. So far, these approaches have suffered from either low efficiency, low bandwidth or additional noise. Here we realize radio-over-fibre qubit readout at millikelvin temperatures. We use one device to simultaneously perform upconversion and downconversion between microwave and optical frequencies and so do not require any active or passive cryogenic microwave equipment. We demonstrate all-optical single-shot readout in a circulator-free readout scheme. Importantly, we do not observe any direct radiation impact on the qubit state, despite the absence of shielding elements. This compatibility between superconducting circuits and telecom-wavelength light is not only a prerequisite to establish modular quantum networks, but it is also relevant for multiplexed readout of superconducting photon detectors and classical superconducting logic.

## Abstract (web summary)

Microwaves are usually used to interact with superconducting qubits, but optical photons can be processed at room temperature. The electro-optical transceiver presented here allows all-optical readout of a qubit without affecting its performance.

## Main

The increasing demand for both higher data transfer rates and energy efficiency has set the path to replacing electrical components by their optical counterparts. This is because of the substantially larger bandwidth of optical signals and the exceptionally low transmission loss in fibres at telecom wavelengths. Recently, this transition affects not only long-distance communication but also short-range links within data centres^1^ or even on a single chip^2^. Moving the processors into a cryogenic environment can decrease the power consumption of computation even further^3^, increase the sensitivity of detection systems^4,5^ and interface classical control systems with cryogenic quantum processors directly^6^. However, such an approach is also susceptible to transmission losses and related heating in electrical wires and thus might also benefit from suitable, low-loss and low-thermal-conductivity optical^7^ or contactless^8^ links.

**Fig. 1:**
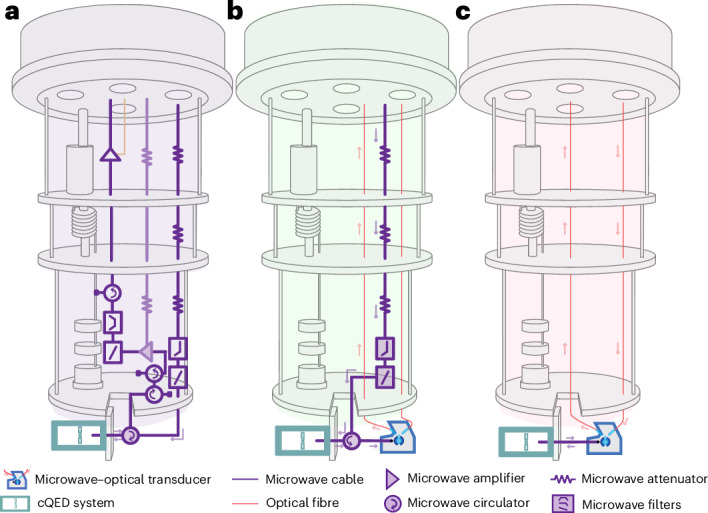
Comparison of conventional and optical qubit readout set-ups in a dilution refrigerator. **a**, Typical microwave in–microwave out set-up consisting of carefully thermalized coaxial cables, attenuators, filters, circulators, a driven parametric amplifier (faded) and a d.c.-biased high-electron-mobility-transistor amplifier, all of which are approximately wavelength sized (centimetres). Note that the components are inserted above the respective temperature stage to make the illustration more compact. **b**, Reduced microwave in–optics out readout set-up replacing the microwave output components with an optically driven, resonant EO transducer. **c**, All-optical, optics in–optics out circulator-free qubit readout based on simultaneous microwave downconversion and upconversion of an optical carrier. Here, all cryogenic microwave components are replaced by a single EO transceiver.

Quantum processors, such as superconducting platforms that operate at ultralow temperatures of a few millikelvin, have particularly demanding input–output requirements. In stark contrast to classical processors, herein the number of external control and readout lines scales linearly with the number of qubits. Currently, the most powerful quantum processors utilize more than 100 qubits requiring hundreds of high-bandwidth coaxial cables with appropriate signal conditioning^9^, that is, attenuation and careful thermalization on the input as well as isolation and low noise amplification on the output (Fig. 1a). Considering the limited cooling power of dilution refrigerators, this architecture might allow thousands of qubits^10^ given that advanced multiplexing strategies are employed^11–13^. This is, ignoring space and financial constraints, still orders of magnitude lower than the millions of qubits expected to be required for fault-tolerant universal quantum computing^14–17^.

Searching for ways to overcome these barriers, photonic links^18–22^ were identified as a promising alternative to conventional^10^, cryo-complementary metal–oxide–semiconductors (cryo-CMOS)^6^ or single flux quantum control^23^ of cryogenic quantum computing platforms. The first optical interconnect with a superconducting qubit detected the average optical power emitted from the qubit, a method that could, at increased efficiency, generate distributed qubit entanglement by means of optical interconnects. From the perspective of the realized qubit-state readout, it was a destructive measurement that prevented further use of the qubit state^24^. Low back-action qubit readout has also recently been shown with a mechanically mediated electro-optical (EO) transducer with record high microwave–optical conversion efficiency^25^ in a scheme comparable to Fig. 1b, but this relatively low bandwidth method necessitates additional microwave pumps with the associated heat load and isolation requirements. Ultrahigh bandwidth readout of an electromechanical system has been demonstrated with a commercial EO modulator operated at 4 K but with limited efficiency and noise performance^19^. On the input side, high-speed and wide-band photodetectors have been used to demodulate microwave control and readout signals^20^. This is a promising approach for multiplexed control but is necessarily dissipative and does not allow the conversion of the readout signals back to the optical domain.

In this Article, we demonstrate all-optical single-shot readout of a superconducting qubit: that is, we replaced both the input and output signal path by one optical fibre each (Fig. 1c). Using a single EO transceiver, that is, a triply-resonant whispering gallery mode single-sideband transducer^26–29^, we simultaneously modulated and demodulated the optical carrier at millikelvin temperatures. This allowed a new circulator-free readout that was used for time-domain characterization of a superconducting transmon qubit enclosed in a three-dimensional superconducting cavity (qubit-cavity system)^30^. The latter was directly connected to the EO transducer by means of a short coaxial cable without the need for any other passive or active cryogenic microwave components. The ability to perform both microwave and optical measurements allows one to make a quantitative comparison of the qubit state assignment fidelity of different readout types. It also enables sensitive Josephson parametric amplifier (JPA) measurements in the presence of the readout laser. We employed that to carefully quantify, within the sensitivity given by the intrinsic loss channels of our devices, the potential radiation and average thermal impact on the mode occupancy and coherence of a superconducting processor that is operated with laser light using modular EO transducers.

## Dynamics and fidelity of conventional and optical readout

**Fig. 2:**
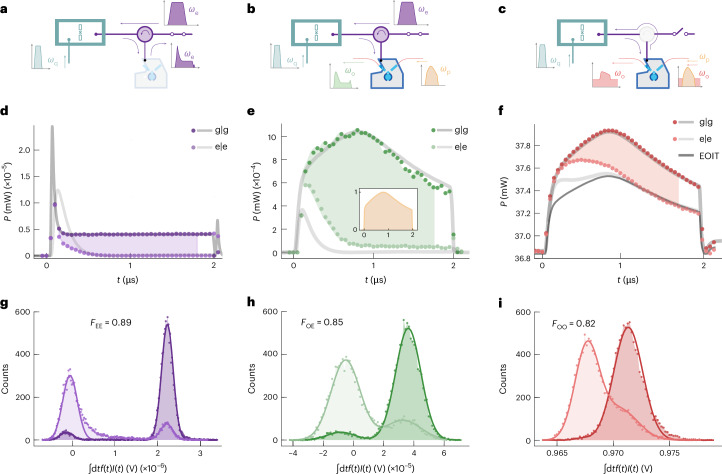
Conventional and optical single-shot readout of a superconducting qubit. **a**–**c**, Sketches of the different readout schemes involving a microwave cavity with bare resonance frequency *ω*_c_ dispersively coupled to a transmon qubit (qubit-cavity system in jade) and the EO transducer, consisting of a second microwave cavity (blue-grey) at *ω*_e_ = *ω*_c_ coupled to an optical whispering gallery mode resonator (light blue). The qubit state was prepared by means of a separate port at *ω*_q_. The EO transducer was operated with an optical pump pulse at *ω*_p_ to parametrically enhance the interconversion of microwave *ω*_e_ and optical *ω*_o_ signals. Conventional microwave readout: a microwave pulse probed the qubit-cavity system and was detected by means of microwave heterodyne detection (**a**). Optical detection of a microwave readout tone: the microwave pulse reflected from the qubit-cavity system was upconverted to the optical domain and detected with optical heterodyne detection (**b**). All-optical readout: a modulated optical carrier was converted to the microwave domain to probe the qubit-cavity system. Its reflection was simultaneously converted back to the optical domain and detected with an optical heterodyne set-up (**c**). **d**–**f**, Averaged time traces (**d**), (**e**) and (**f**) of the measured heterodyne signal powers corresponding to the readout schemes shown in **a**, **b** and **c** postselected on successful measurements of the prepared qubit state (g∣g and e∣e) based on 15,000 independent trials. Grey lines show theoretical predictions which are expected to deviate for $\left\vert \rm{e}\right\rangle$ before the steady state is reached (see text and Supplementary Information). The shaded areas highlight the difference between both qubit-state responses, for the interval where we extract the weighting functions *f* = *I*_e_ − *I*_g_ for the temporal in-phase quadrature integration. The inset in **e** is a normalized measurement of the optical pump power. For reference, panel **f** also shows the simulated optical response of the EO transducer without the reflection from the qubit-cavity system, that is, only due to electro-optically induced transparency (EOIT). **g**–**i**, Histograms of 15,000 single shots obtained by integrating the weighted in-phase quadrature *f*(*t*)*I*(*t*) shown in **g**, **h** and **i** with state assignment fidelities ${{\mathcal{F}}}_\mathrm{EE}\text{,}\,{{\mathcal{F}}}_\mathrm{OE}\, \text{and}\, {{\mathcal{F}}}_\mathrm{OO}$, corresponding to the readout schemes **a**, **b** and **c**.

We start with a comparison of the three different readout methods schematically depicted in Fig. 1 with the relevant components at different temperature stages (see Supplementary Information for the detailed experimental set-up), and which is illustrated in more detail with the main components at the coldest stage of the cryostat in Fig. 2a–c, including the corresponding pulse shapes of the respective input and output tones and the signal paths: (1) all-microwave readout (Figs. 1a and 2a) with a microwave tone sent through coaxial cables to the qubit-cavity system and detected with a standard microwave heterodyne set-up; (2) microwave–optical readout with optical detection of the same microwave signal as in (1) from the qubit-cavity system after using it for the modulation of laser light by means of the EO transducer (Figs. 1b and 2b); and (3) all-optical readout. We sent modulated light to the EO transducer. The demodulated microwave pulse entered the qubit-cavity system and its reflection was converted back into the optical domain using the same EO transducer before being analysed with an optical heterodyne detector at room temperature (Figs. 1c and 2c). All three schemes can be realized without set-up changes except for the state of a cryogenic radiofrequency (RF) switch, as shown in Fig. 2a–c. While the first two methods can be performed simultaneously, opening the RF switch for the all-optical readout effectively removes the circulator. This prevents the optically demodulated microwave signal from entering the microwave output line.

The operation frequency of the EO transducer was determined by its optical free-spectral range *ω*_FSR_/(2π) = 8.8065 GHz, which was set by the diameter of the lithium niobate whispering gallery mode resonator. To achieve a triply-resonant configuration that maximized the transduction efficiency, we tuned the EO microwave cavity in resonance *ω*_e_ = *ω*_FSR_ (ref. ^26^). Similarly, to maximize the dispersive qubit readout efficiency^31^ we also tuned the qubit cavity’s bare resonance to the same frequency *ω*_c_ = *ω*_FSR_. Both tunings were implemented with a piezoelectric actuator.

The transmon qubit with anharmonicity *ν*/(2π) = −201 MHz was alternately prepared in its first excited state $\left\vert \mathrm{e}\right\rangle$ or thermalized in its ground state $\left\vert \mathrm{g}\right\rangle$ by selectively applying a flat-top-Gaussian microwave pulse of duration 104 ns at the qubit transition frequency *ω*_q_/(2π) = 6.251 GHz by means of a dedicated drive line (Fig. 2a–c). The readout tone, on the other hand, was either applied by means of filtered and attenuated input coaxial lines (Fig. 2a–b) or directly generated by the EO transducer (Fig. 2c) by means of resonantly enhanced optical downconversion^32^. The readout amplitude corresponding to approximately $\sqrt{{n}_{{\rm{meas}}}}=122\,{{\rm{photons}}}^{1/2}$ in the cavity was chosen to optimally benefit from the Jaynes–Cummings nonlinearity of the qubit–cavity system^33,34^ that maps the qubit-state-dependent dispersive frequency shift of the resonator *χ*/(2π) = 3.3 MHz into a large readout amplitude difference at the bare qubit cavity frequency (see Supplementary Information for details). Although this is not quantum-non-demolition (QND) in character, the latter enables single-shot qubit readout with an increased signal-to-noise ratio^35^ and thus allowed us to omit the parametric amplifier in Fig. 1a.

Figure 2d shows the averaged reflected amplitude in power units postselected on measuring the prepared state from heterodyne detection for the all-microwave readout. The measured dynamics with the qubit initialized in its ground state is in excellent agreement with the input–output relationships of the transducer microwave cavity reflection alone (dark grey line), which reveals that the qubit cavity did not exhibit a resonance at the readout frequency *ω*_e_. By contrast, when the qubit was prepared in the excited state, the qubit cavity resonance appeared at the bare resonance frequency with *ω*_c_ ≈ *ω*_e_ and the reflected power decreased (light grey line). Although the initial dynamics are out of reach to be modelled given the high photon numbers, we adopted a simple cascaded cavity model between the qubit cavity and the EO microwave cavity^36^ (see Supplementary Fig. 1 for more details). This accurately predicts the steady-state result after times >1.0 μs without free parameters (light grey) and, consequently, the readout contrast between both states. We then used these averaged measurements to optimize the quadrature rotation and the integration weights as the difference between the response of both states (shaded region in Fig. 2d) to maximize the distinguishability for the single-shot readout.

The corresponding single-shot histograms from 1.5 × 10^4^ independent measurements for each qubit state are shown in Fig. 2g with double-Gaussian fits to extract the relevant errors^37^. The maximum state assignment fidelity of ${{\mathcal{F}}}_\mathrm{EE}=1-\left(P(\mathrm{e}| \mathrm{g})+P(\mathrm{g}| \mathrm{e})\right)/2=0.89\pm 0.01$ was reached after an integration time of 1.8 μs, with *P*(*x*∣*y*) being the probability of measuring the qubit in state $\left\vert x\right\rangle$ after preparation of state $\left\vert y\right\rangle$. The clear separation between the two distributions indicates a negligible overlap error (*ϵ*_ol,EE_ < 10^−10^). The ground state error (*ϵ*_g,EE_ ≈ 7%) originated partly from thermal excitation (1.5% as quantified below), whereas the rest was attributed to transitions induced by the comparably long high-power readout pulse^38^. The excited state readout resulted in an error of *ϵ*_e,EE_ ≈ 16%. Interestingly, the asymmetric tail in the excited state Gaussian towards the ground state distribution originated from switching before steady state was reached and not from qubit decay due to the limited coherence as in the low-power limit (Supplementary Information).

For a direct comparison, we simultaneously also read out a small part of the reflected microwave readout tone optically (Fig. 2b). After resonantly enhanced microwave-to-optical conversion^32^, in which about 3% of the intracavity microwave photons were converted, we performed optical heterodyne detection, which yielded the averaged time traces shown in Fig. 2e. In comparison with the microwave readout, we found slower dynamics due to the limited conversion bandwidth of ~10 MHz for the optical readout signal at *ω*_o_/(2π) = 193.4 THz. In addition, the shape of the optical pump pulse with peak power ~140 mW at frequency *ω*_p_ = *ω*_o_ − *ω*_FSR_ (inset in Fig. 2e) was imprinted on the optical readout signal because it parametrically enhanced the microwave–optical transduction in very good agreement with theory for the ground state (dark grey line). We attribute the deviation of the steady-state coherent power for the excited state (light grey line) to imperfections in our optical phase correction at lower optical powers. The separation between the single-shot state distributions decreased (Fig. 2h) compared to the all-microwave readout, resulting in a larger overlap error of *ϵ*_ol,EO_ = 2% and a slightly reduced microwave–optical state assignment fidelity of ${{\mathcal{F}}}_\mathrm{OE}=0.85\pm 0.01$.

Finally, also in case of the all-optical readout, the optically demodulated microwave tone (corresponding to $\sqrt{{n}_{{\rm{meas}}}}=116\,{{\rm{photons}}}^{1/2}$ in the qubit cavity) resulted in well-distinguished state dependent trajectories, as shown in Fig. 2f. The large optical background signal was due to the cumulative reflection of the optical input, for example, at the coupling prism. The bandwidth of the EO transducer now also slowed down the dynamics of the build-up of microwave readout photons. Additionally, electro-optically induced transparency^39^ raised the signal levels during the optical pulse, visualized as the simulated optical reflection of this EO transducer if there was no qubit-cavity system connected (dark line, EOIT). Altogether, this led to excellent agreement between the measured data and theory (light grey and grey lines). The moderate reduction of fidelity ${{\mathcal{F}}}_\mathrm{OO}=0.82\pm 0.01$ can be fully attributed to the larger overlap error between the state distributions shown in Fig. 2i. This result proves the feasibility of an isolator-free qubit readout without cryogenic microwave components.

## Time-dependent qubit measurements

**Fig. 3:**
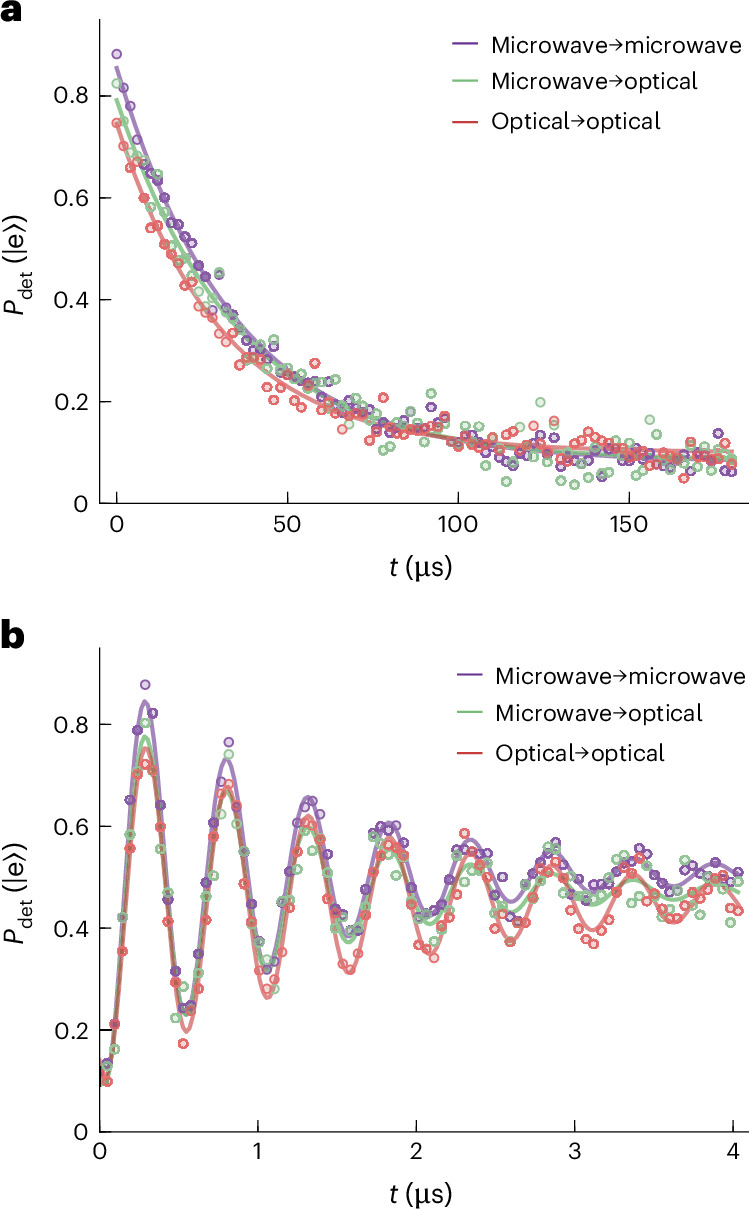
Qubit coherence for different readout methods. **a**, Measured excited state detection probability ${P}_{\det }(\left\vert \rm{e}\right\rangle )$ after a π pulse for varying measurement delays *t* using the three different readout methods shown in Fig. 2. **b**, Measured Ramsey oscillations using two π/2 pulses separated by a variable delay *t* and detuned by ~2 MHz from the qubit transition for the three readout methods.

We used all three readout methods to extract the longitudinal *T*_1_ and transverse relaxation time ${T}_{2}^{\,* }$ of the superconducting qubit, based on a 10 Hz repetition rate for five individual measurements averaged over 1 h each to be insensitive to short-term fluctuations in the coherence time yielding a consistent *T*_1_ of 34.4 ± 1.6 μs (microwave readout), 35.9 ± 5.0 μs (microwave–optical) and 31.9 ± 9.9 μs (all-optical). Error bars are the two-sided 90% confidence interval of the mean from a Student's *t* distribution owing to the low sample size (five). Figure 3a shows the energy relaxation averaged over all five measurements for the three types of readouts. The observed differences are within the observed *T*_1_ variability as the measurements were taken on different days. The slightly reduced contrast was expected due to the previously extracted ${{\mathcal{F}}}_{ij}$.

**Fig. 4:**
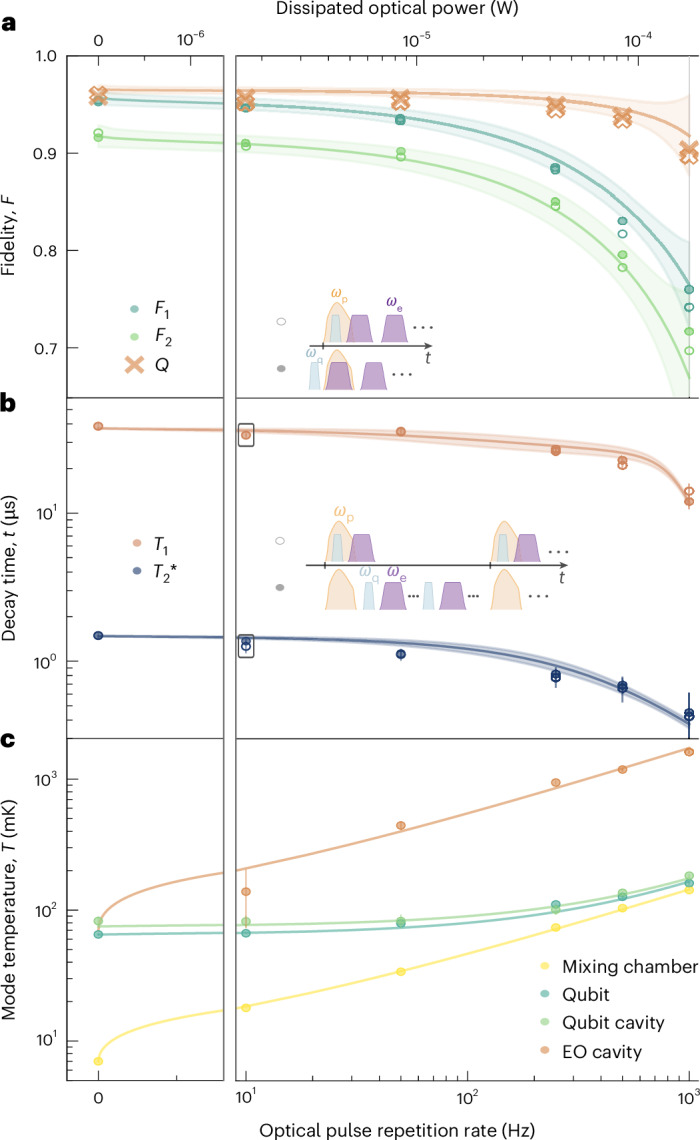
Impact of the optical pump. **a**, Measured state assignment fidelities ${{\mathcal{F}}}_{1}$ and ${{\mathcal{F}}}_{2}$ of two consecutive JPA-assisted microwave measurements (filled circles) and corresponding QND metric (${\mathcal{Q}}$, crosses) obtained in the presence of a 2-μs-long optical pump pulse of ~0.14 W applied during the first readout as a function of repetition rate and calculated dissipated optical power (top axis) together with theory (lines and 3*σ* confidence bands). Approximately $1-{(1-2{\eta }_{\rm{o}})}^{2}\approx 69 \%$ of the average optical power sent to the sample was dissipated in the device. Empty circles (mostly overlapping with filled circles) denote measurements for which the optical pump was applied also during state preparation. Data are represented as mean ±3*σ* but error bars are smaller than the marker size. The insets show pulse sequences for the differently triggered measurements. Qubit preparation, readout and optical pump are denoted by *ω*_q_, *ω*_e_ and *ω*_p_, respectively. **b**, Measured qubit coherence times $({T}_{1},\,{T}_{2}^{\,* })$ when the optical pulse was synchronized with each qubit preparation and readout pulse (empty circles) and for a free-running measurement sequence (filled circles) versus optical pulse repetition rate. Squares indicate the mean of the optical readout results in Fig. 3. The decrease in *T*_1_ and ${T}_{2}^{\,* }$ was accurately modelled with theory (red and blue line with 3*σ* confidence band), based on the measured thermal occupancy shown in **c**, the expected quasiparticle distribution and Purcell decay (red line). Data error bars show the two-sided 90% confidence interval of the mean according to a Student *t* distribution for five measurements (compare with Fig. 3). **c**, Measured temperature of the mixing chamber plate (yellow dots) and the different microwave modes (dots) together with power law fits as a guide to the eye. Mean values and error bars stem from the respective fits with 3*σ* confidence bands and corresponding error propagation calculations.

Similar conclusions can be drawn from the measured exponential decay of the Ramsey oscillations shown in Fig. 3b. The fitted mean transverse decays ${T}_{2}^{\,* }$ for all three measurements, 1.3 ± 0.1 μs, 1.4 ± 0.3 μs and 1.7 ± 0.1 μs, are comparable. The all-optical readout yielded the longest coherence. The comparably low ${T}_{2}^{\,* }$ was limited by shot noise from residual thermal cavity photons^40,41^ owing to the strong qubit–cavity coupling and small detuning (see discussion below and Fig. 4). We attribute the measured deviation from the expected theoretical limit of ${T}_{2,\rm{max}}^{\,* }\approx 4\,\upmu{\rm{s}}$ to fabrication and design-related issues, as a Hahn-echo measurement of *T*_2,echo_ = (1.40 ± 0.06) μs, 2*σ* fit confidence interval, excluded a low frequency noise origin. Moreover, we observed the same coherence times when the readout laser was turned off, or when the optical pulse was applied during the qubit state preparation, as discussed below and in Fig. 4. Our measurements, therefore, clearly demonstrate the integrity of superconducting qubit coherence using a photonic readout.

## Quantifying the impact of optical absorption heating

Although the previous measurements have shown that reliable qubit characterization is feasible with a strong optical readout pulse, a more sensitive method is required to fully quantify the potential radiative^42,43^ and thermal^27^ impact of high-energy pump photons. In the following, we use a near-quantum-limited non-degenerate JPA^44^ to perform a standard, non-destructive microwave readout in the dispersive low-power regime to quantify such effects.

First, we performed two consecutive readouts of the qubit after it was prepared in either the ground or excited state. The first readout pulse was applied in the presence of the previously used optical pump pulse. For comparison, we also measured this sequence when the laser was off. Figure 4a shows the extracted qubit state assignment fidelity ${{\mathcal{F}}}_{1(2)}$ of the first (second) measurement in cyan (green) for increasing optical pulse repetition rates. From that, it was instructive to calculate the average optical power that was dissipated at the mixing chamber stage of the dilution unit due the intrinsic cavity loss and imperfect mode overlap with the optical fibre (top axis). The observed dependence was in excellent agreement with theory (lines and 3*σ* confidence bands) for spontaneous emission scaling with $1-{\rm{e}}^{-t/{T}_{1}}$ and the independently measured thermal excitation of the qubit (Fig. 4c). The remaining discrepancy was fitted to be ≤1% and attributed to either measurement (or optical radiation) induced transitions or state preparation errors.

The QND metric is defined as the fraction of measurements where two consecutive readouts yield the same qubit state^45^, that is, ${\mathcal{Q}}=\left(P(\mathrm{g}_{{\rm{2}}}| \mathrm{g}_{1})+P(\mathrm{e}_{{\rm{2}}}| \mathrm{e}_{1})\right)/2$, and therefore probes the impact of an applied readout tone. Importantly, ${\mathcal{Q}}$ (orange) was comparable for moderate repetition rates and a dark measurement without laser light, which implies a minimal (if any) direct impact of the optical pulse on the qubit. This interpretation is supported by additional measurements for which the same optical pulse was applied also during the qubit state preparation (empty circles in Fig. 4a), which mostly overlap with the filled circles (compare with pulse sequence in inset). Measurements are in exellent agreement with the theoretical dependence of ${\mathcal{Q}}$ (orange line) based on temperature dependent spontaneous qubit emission.

Figure 4b shows coherence times as a function of optical pulse repetition rate with optical pulses sent simultaneously with qubit preparation and the readout pulses (empty circles) together with free-running measurements for which the optical pulse was not synchronized with the microwave measurements (~5 kHz repetition rate, filled circles). The latter method is insensitive to instantaneous radiation-based impact, for example, by means of the generation of quasiparticles. The very close agreement between the two types of measurements, the small difference between low repetition rates and laser-off measurements, and the very good agreement with the coherence times obtained with the (all-)optical readout of Fig. 3 (black squares) indicate the absence of such radiative effects.

The measured increase in the longitudinal decay rate *γ*_1_ = 1/*T*_1_ as a function of applied average optical power compared with the ‘cold’ decay rate without laser light, ${\gamma }_{1}^{0}$, was in excellent agreement with a prediction of thermal effects due to optical absorption heating in the EO transducer,

1 $${\gamma }_{1}={\gamma }_{1}^{0}\left(1+2{n}_{{\rm{th}}}+\frac{\sqrt{2\uppi {k}_{{\rm{B}}}{T}_{{\rm{q}}}/{\varDelta }_{{\rm{sc}}}}}{{x}_{{\rm{qp}}}^{0}}{\rm{e}}^{-\frac{{\varDelta }_{{\rm{sc}}}}{{k}_{{\rm{B}}}{T}_{{\rm{q}}}}}\right)+\frac{{g}_{{\rm{qc}}}^{2}}{{\varDelta }_{{\rm{qc}}}^{2}}{\varDelta }_{{\kappa }_{{\rm{c}}}},$$

where *k*_B_ is Boltzmann's constant, as shown in Fig. 4b (red line and 3*σ* confidence band). Equation (1) takes into account only direct qubit excitation from black body radiation *n*_th_ (ref. ^46^) at temperature *T*_q_, thermal equilibrium quasiparticles with a superconducting gap *Δ*_sc_ of 205 μeV as well as a typical non-equilibrium quasiparticle density of ${x}_{{\rm{qp}}}^{0}=1.6\times 1{0}^{-7}$ (ref. ^47^), and an increase in the Purcell rate with qubit–cavity coupling *g*_qc_ and detuning *Δ*_qc_. Using the independently measured qubit temperature *T*_q_ shown in Fig. 4c and measurements of the slightly broadened qubit cavity linewidth ${\varDelta }_{{\kappa }_{{\rm{c}}}}={\kappa }_{{\rm{c}}}-{\kappa }_{{\rm{c}}}^{0}$ of up to 240 kHz at higher repetition rates (temperatures), ${\gamma }_{1}^{0}=37\,\upmu s$ remained as the only fit parameter.

In a similar manner, the relative dependence of the transverse decay ${T}_{2}^{\,* }$ (Fig. 4b, blue) was fully consistent with the increased dephasing rate from thermal photon shot noise due to the rising qubit cavity temperature (Fig. 4c) and the increase in *γ*_1_ as described above. *T*_2,echo_ and ${T}_{2}^{\,* }$ show again no measurable difference. Quasiparticles are also not believed to have a dominating effect on dephasing in transmon qubits^48–50^.

Finally, we investigated the average temperature distribution of the different components, which is used for the theory in Fig. 4a,b. Figure 4c shows the measured base plate temperature from a calibrated rutenium oxide sensor, as well as the mode temperature of the superconducting qubit as obtained from thermally excited $\left\vert \rm{e}\right\rangle \leftrightarrow \left\vert\,\rm{f}\,\right\rangle$ Rabi oscillations^51^. The temperature of the qubit cavity was extracted from populated Ramsey oscillations^52^, and the EO microwave cavity temperature was calculated from the measured power spectral density at its output^27^. These measurements were performed free running but with the same optical pulse applied to the transducer.

When the laser was off, all components thermalized to a temperature of ~75 mK, whereas the refrigerator reached a base temperature of ~7 mK (Fig. 4c). When the optical pump was on, it acted as a localized heat source that increased the EO microwave mode temperature (orange). The measured dependence on the time-averaged applied optical power of $\propto \,{\bar{P}}_{{\rm{opt}}}^{0.54}$ agreed with previous findings for continuous-wave optical pump experiments^27^. The EO transducer was in very good thermal contact with the refrigerator’s base plate, which heated up the refrigerator with the same power law (yellow) fundamentally originating from the dependence of the mixing chamber cooling power, *P*_MXC_, on its temperature, *T*_MXC_, that is, $\sqrt{{P}_{{\rm{MXC}}}}\propto {T}_{{\rm{MXC}}}$ (ref. ^53^). The resilience of the qubit-cavity system to radiation and heating at moderate repetition rates (Fig. 4a,b) was reflected again in the mode temperature of the qubit and the dispersively coupled cavity. Their temperature increased only slightly compared with the laser-off situation for moderate repetition rates. One reason for this behaviour was the detuning between the transducer cavity mode and the qubit-cavity system by the Lamb shift *χ*_0_/(2π) = 26 MHz, except for the moment when the high-power readout pulse was applied. Other reasons were the careful thermalization of all components and the large heat capacity and thermal contact area of the bulk EO transducer compared with integrated photonics approaches. However, as the qubit-cavity system was thermally connected to the mixing chamber as well, its mode temperature rose as soon as the fridge temperature approached the thermalization temperature of the qubit cavity (cyan and light green). This behaviour was consistent with the sharp decline in the qubit coherence and readout fidelity for higher repetition rates in Fig. 4a,b.

## Conclusions and prospects

One of the main motivations for this work was to simplify the cryogenic measurement set-up by eliminating bulky and costly microwave components that are the source of a substantial heat load^10^. By contrast, even the smallest cooling power at the mixing chamber plate can handle the passive heat load of millions of fibres^20^ and their small cross-section mitigates the problem of space constraints raised by millimetre-sized coaxial cables. Nevertheless, the active heat load of this proof-of-principle all-optical readout limits the duty cycle and prevents a direct scaling-up to many readout-out lines. In the present case, owing to our low optical coupling efficiency of *η*_o_ = 0.22, a majority of the parametric pump power was absorbed at the mixing chamber, leading to the observed temperature increase associated degradation of the qubit coherence shown in Fig. 4. Therefore, in the future, the optical coupling efficiency is a critical parameter to improve, and optimized devices will also need to out-couple the majority of the reflected light to avoid absorption in the refrigerator. Similarly, the power efficiency is another critical parameter that can be improved dramatically, for example with integrated photonic devices. Examples are electro-optomechanical devices yielding cooperativities *C* ≈ 1 for one billion times lower optical pump power^25,54^ than that used in this work, albeit with lower bandwidth and, in the case of the latter, increased noise. The aforementioned improvements are necessary to ultimately gain, in addition to the drastic set-up simplifications, the heat load advantage compared with standard microwave cabling or the use of cryogenic photodetectors^20^. Although the latter fundamentally generate a heat load on the dilution unit, the dissipation of the readout presented here depends only on the efficiency and, hence, is subject to device engineering.

One of the limitations of the implemented optical readout is the need for a comparably large number of readout photons *n*_meas_. Scaling the histograms in Fig. 2g–i with the corresponding readout amplitude $\sqrt{{n}_{{\rm{meas}}}}$ yielded the quantum efficiency ${\eta }_{\det }={\sigma }_{0}^{2}/{\sigma }_{\det }^{2}$ with the Gaussian variance of the measured histogram ${\sigma }_{\det }^{2}$ and the variance of an ideal phase insensitive amplifier ${\sigma }_{0}^{2}=0.5$ (ref. ^55^). For the conventional microwave readout (without JPA) we extracted *η*_det,EE_ ≈ 1.3 × 10^−3^. This is consistent with a comparably large amount of loss between the qubit-cavity system and the first amplifier (transmission of only <3%) due to the extra circuit elements such as the EO transducer with reflectivity ${(1-2{\eta }_{\rm{e}})}^{2}=0.09$, with the microwave coupling efficiency *η*_e_. On the other hand, for the two optical readouts, we found ${\eta }_{\det ,{\rm{OE}}}\approx {\eta }_{\det ,{\rm{OO}}}\approx 1.5\times 1{0}^{-4}$, which agrees with the moderate total EO device conversion efficiency *η*_eo_ = 0.3% and optical losses. This is within an order of magnitude of a recent experimental result with an electro-optomechanical system using a longer (15 μs) readout pulse^25^, which achieved a readout efficiency of up to 8 × 10^−4^. Importantly, even with just the original performance of this device^32^ (we observed a degradation of the intrinsic optical *Q*-factor in repeated cooldowns) a QND single-shot readout without electronic amplifiers and readout times of ~1 μs would be within reach. With further realistic improvements of coupling and transmission losses, close to quantum limited detection efficiencies will be possible. This is relevant for photonic RF sensing^56^ as well as for high-bandwidth and high-fidelity qubit readout comparable to the state of the art^37^. Finally, we want to emphasize that the same device operating at the qubit transition frequency *ω*_q_ could also be used for qubit control with a π pulse length of about 110 ns. Shorter pulses would be limited by the bandwidth of the current device.

In summary, we have demonstrated a circulator-free superconducting qubit readout with an all-optical scheme that relies only on optical (de-)modulation and optical heterodyne detection. Such a platform offers a substantially simplified cryogenic set-up in which signal conditioning is performed at room temperature and optical fibres act as link to the cryogenic environment. Somewhat surprisingly, we found that comparably high-power optical pulses in the 100 mW range with low duty cycle do not have a detrimental effect on the qubit coherence, despite the absence of shielding elements. This result, when combined with recent integrated photonics demonstrations of more power-efficient and higher repetition rate optical control^57^ and readout^58^ of planar superconducting qubits, provides a viable path towards all-integrated photonic operation of superconducting quantum processors.

## Online content

Any methods, additional references, Nature Portfolio reporting summaries, source data, extended data, supplementary information, acknowledgements, peer review information; details of author contributions and competing interests; and statements of data and code availability are available at 10.1038/s41567-024-02741-4.

## Supplementary information

Supplementary Information Supplementary Figs. 1–3, Discussion and Tables 1–2.

## Data Availability

The data used to produce the plots in this paper are available via Zenodo at 10.5281/zenodo.14033026 (ref. ^59^).

## Code availability

The code used to produce the plots in this paper is available via Zenodo at 10.5281/zenodo.14033026 (ref. ^59^).
