# Supplementary material for: All-optical superconducting qubit readout
Source: Nat Phys. 2025 Feb 11;21(3):393–400. doi: 10.1038/s41567-024-02741-4 (PMC11908971; doi:10.1038/s41567-024-02741-4)
Supplement: Supplementary file 1 — Supplementary Figs. 1–3, Discussion and Tables 1–2. [file 41567_2024_2741_MOESM1_ESM.pdf]

nature physics

Supplementary information

https://doi.org/10.1038/s41567-024-02741-4

All-optical superconducting qubit readout

In the format provided by the authors and unedited

CONTENTS

Page

I. Jaynes-Cummings nonlinearity readout and toy model 3
- A Experimental observation . . . 3
- B Microwave readout model . . . 3
- C Microwave-optical readout model . . . 4
- D Full optical readout model . . . 4
- E Skewed excited state distribution . . . 4

II. Fabrication 5
- A The cQED system . . . 5
- B The electro-optic transceiver . . . 5

III. Experiment 6

References 7

Introduced in Main Text	
C	cooperativity ($C = 4g^2/\kappa_e\kappa_o$)
$\chi_{\mathrm{g,e}}$	Dispersive qubit-state-dependent frequency shift
χ_0	Lamb shift
$\Delta_q c$	Detuning between the transmon qubit and the superconducting cavity of the cQED system
$\Delta_s c$	Superconducting gap
$\epsilon_{\mathrm{g,EE}}, \epsilon_{\mathrm{e,EE}}$	Ground state, excited state error
$\epsilon_{\mathrm{ol,EE}}, \epsilon_{\mathrm{ol,OE}}$	Overlap error for mw, mw→opt readout
$\eta_{\mathrm{det,EE}}, \eta_{\mathrm{det,OE}}, \eta_{\mathrm{det,OO}}$	Quantum efficiency for microwave, microwave→optical, optical detection
η_e	External coupling efficiency transducer cavity
η_{eo}	Total electro-optic conversion efficiency
η_o	External coupling efficiency optical resonator
$f(t)$	Weighting function for time-trace integration
$\mathcal{F}_{\mathrm{EE}}, \mathcal{F}_{\mathrm{OE}}, \mathcal{F}_{\mathrm{OO}}$	Assignment fidelity for mw→mw, mw→opt, and opt→opt readout
$\|g\rangle, \|e\rangle, \|f\rangle$	Qubit ground, excited, second excited state
$I(t)$	In-phase quadrature of the microwave output field
mw → mw,mw → opt,opt → opt	Sending and measuring microwaves, sending microwaves and measuring optics, sending and measuring optics
$\sqrt{n_{meas}}$ / n_{meas}	readout amplitude / readout photon number in the cQED cavity
n_{th}	Mean photon number $(e^{\hbar\omega/k_BT} - 1)^{-1}$ of radiator at temperature T and frequency ω
ν	Qubit level anharmonicity
ω_c	Resonance frequency of the cQED system
ω_{FSR}	Optical free spectral range
ω_e	Microwave resonance frequency of the electro-optic transducer
ω_o	Optical signal frequency
ω_p	Optical pump frequency
ω_q	First qubit transition frequency
$P(\mathrm{e}\|\mathrm{g})$ $(P(\mathrm{g}\|\mathrm{e}))$	Probability for assigning $\|e\rangle(\|g\rangle)$, after preparing $\|g\rangle(\|e\rangle)$
$P(e_1\|e_2)$ $(P(g_1\|g_2))$	Probability to measure $\|e\rangle(\|g\rangle)$ in two successive measurements
P_{opt}	Optical power
$\mathcal{Q}$	Quantum non-demolition metric (QND-ness)
σ^2_{det}	Scaled Gaussian variance
σ^2_0	Variance ideal amplifier
T	Mode temperature
T_1	Energy relaxation
$T_{2,echo}$	Transverse echo relaxation
T_2^*	Transverse decay
**Introduced in Supplementary information**	
$\hat{a}_j$	Mode, $j \in (e, o, c, p, s, tm)$
$\hat{a}_{j,\mathrm{in}}$	Input field (noise) operator for the microwave and optical mode, $j \in (e, o, c)$
$\hat{a}_{j,out}$	Readout field, $j \in (e, o, c)$
δ_j	Detuning, $j \in (o, s, tm)$
η_j	External cavity coupling efficiency of individual mode, $j \in (e, o, c, p)$
$\eta_{j,i}$	Coupling efficiency from i to j, $i, j \in (e, o, c)$
f_{bare}, ω_{bare}	Bare resonator frequency cQED, $2\pi f_{bare}$
g	Photon enhanced electro-optical coupling rate ($g = \bar{a}_p g_0$)
g_0	Electro-optic vacuum coupling rate ($g = \bar{a}_p g_0$)
g_{qc}	qubit-cavity coupling rate in the cQED system
J	Coupling rate between the optical Stokes mode and TM mode
κ_j	Total loss rate of individual mode, $j \in (e, o, c, s, p)$
$\kappa_{j,ex}$	External loss rate of individual mode, $j \in (e, o, c)$
m	Microwave azimuthal mode number, $m = 1$
P_{ee}	Detected power for mw→mw
$\hat{\sigma_z}$	Pauli operator
τ	Delay
x^0_{qp}	Density of non-thermal-equilibrium quasiparticles per Cooper pair

Supplementary Tab. 1. List of variables.

I. JAYNES-CUMMINGS NONLINEARITY READOUT AND TOY MODEL

A. Experimental observation

In the dispersive limit, the nonlinearity from the Jaynes-Cummings interaction can be employed for a measurement of the qubit state with high signal-to-noise ratio [1]. The measurement effectively makes use of the qubit-induced cavity anharmonicity (Lamb shift $\chi_0/2\pi = 26$ MHz) which depends on the cavity drive power for higher occupations and the dispersive shift of the cavity at low drive powers ($\chi/2\pi = 3.3$ MHz). The combination of both effects leads to the shift of the cavity resonance to its bare frequency $f_{bare} = 8.806$ GHz at different drive powers for different qubit states. This allows a qubit state-dependent readout with high SNR (cf. dashed lines in Fig. 1a and b). Theoretical descriptions of this behavior include either higher qubit levels [2] or a semi-classical treatment for large cavity-qubit detunings and drive power-dependent anharmonicities [3]. Both models led to qualitative agreement of the frequency spectrum with the experimental observation. Phenomenologically, the situation can be described by the cQED cavity resonance either being at its bare resonator frequency ($|e\rangle$) or being completely off-resonant ($|g\rangle$), as shown in Fig. 1c. The visible resonance for the latter case stems from the microwave cavity of the electro-optic transducer, which is tuned to f_{bare} in our system. Thus, one observes either the transducer cavity alone or being on resonance with the cQED cavity (Fig. 1c). Even though this omits the qubit-cavity interaction and can therefore not predict the temporal dynamics with the qubit being measured in $|e\rangle$, it is sufficient to model the system with independently calibrated parameters in steady state (Fig. 1d).

B. Microwave readout model

We model the microwave system consisting of the cQED cavity and the transducer microwave cavity as two cascaded cavities with reflective ports (coupling $\eta\kappa$) and intrinsic loss rates $(1-\eta)\kappa$. Specifically, the microwave readout field from the cQED cavity $\hat{a}_{c,\text{out}}(t)$ travels through superconducting cables of efficiency $\eta_{e,c}$ and delay τ, and enters the microwave cavity of the electro-optical transducer with $\hat{a}_{e,\text{in}}(t) = \eta_{e,c}\hat{a}_{c,\text{out}}(t+\tau)$ [4].

If we send the specific readout power to the cQED device, at which the Jaynes-Cummings nonlinearity allows to detect the qubit state (Fig. 1c), the cQED cavity is either on-resonant with the transducer cavity ($\omega_e = \omega_c$, excited state) or completely reflective (ground state). We model the latter by a detuning χ_0.

$$\dot{\hat{a}}_e = -\frac{\kappa_e}{2}\hat{a}_e + \sqrt{\eta_e\kappa_e}\eta_{e,c}\hat{a}_{c,\text{out}}, \tag{1}$$

$$\dot{\hat{a}}_c = \left[-i\frac{\chi_0}{2}(\langle\hat{\sigma_z}\rangle + 1) - \frac{\kappa_c}{2}\right]\hat{a}_c + \sqrt{\eta_c\kappa_c}\hat{a}_{c,\text{in}}. \tag{2}$$

We model the system using decoupled equations assuming the signal propagation is unidirectional. This is a reasonable assumption because of the microwave circulator between both cavities. We want to stress again that this is only a phenomenological model to describe the behavior of the system at the specific power chosen for the Jaynes-Cummings readout.

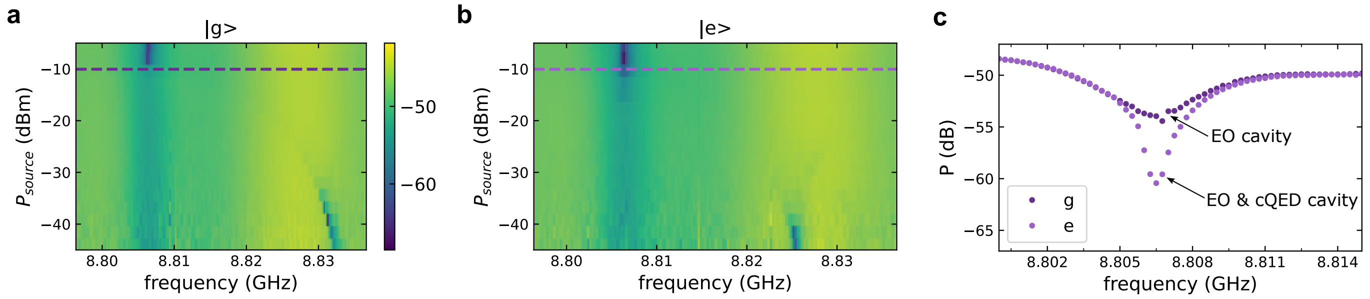

Supplementary Fig. 1. **Jaynes-Cummings readout.** **a**, Microwave reflection spectrum from a power sweep of the cQED and EO cavities with the qubit in ground state, the electro-optic transducer cavity at $\omega_e/2\pi = 8.806$ GHz, and additionally the cQED cavity switched to $\omega_{\text{bare}} = \omega_e$ at high readout powers. **b**, Similar spectrum to **a** with the qubit prepared in the excited state. The increase in contrast as a result from the cQED cavity being resonant with the transducer cavity happens at slightly lower powers than for the ground state which allows for a qubit readout at the chosen power (dashed lines). **c**, Reflection for both qubit states at the microwave readout power for the Jaynes-Cummings readout used in the main text . The contrast difference allows to detect the qubit states at high powers at the bare cQED cavity frequency.

C. Microwave-optical readout model

The microwave-optical readout of the qubit state is enabled by converting the microwave field from the cQED system into the optical domain using the electro-optic transducer. This allows for efficient conversion from microwave to optical fields. The electro-optic device is driven by a resonant optical pump pulse, with dynamics given by,

$$\dot{\bar{a}}_p = \left(i\Delta_p - \frac{\kappa_p}{2}\right)\bar{a}_p + \sqrt{\eta_p\kappa_p}\bar{a}_{p,\mathrm{in}}, \tag{3}$$

with $g(t) = \bar{a}_p(t)g_0$ the cavity enhanced electro-optical coupling rate. The dynamics of the multi-mode electro-optic device can be described by the quantum Langevin equation,

$$\begin{aligned}
\dot{\hat{a}}_e &= -\frac{\kappa_e}{2}\hat{a}_e - ig\hat{a}_o - ig^*\hat{a}_s^\dagger + \sqrt{\eta_e\kappa_e}\hat{a}_{e,\mathrm{in}}, && (4)\\
\dot{\hat{a}}_o &= \left(i\delta_o - \frac{\kappa_o}{2}\right)\hat{a}_o - ig\hat{a}_e, && (5)\\
\dot{\hat{a}}_s &= \left(i\delta_s - \frac{\kappa_s}{2}\right)\hat{a}_s - ig^*\hat{a}_e^\dagger - iJ\hat{a}_{\mathrm{tm}}, && (6)\\
\dot{\hat{a}}_{\mathrm{tm}} &= \left(i\delta_{\mathrm{tm}} - \frac{\kappa_{\mathrm{tm}}}{2}\right)\hat{a}_{\mathrm{tm}} - iJ\hat{a}_s. && (7)
\end{aligned}$$

where $a_{e,in}$ denotes again the output field from the cQED cavity. We note that, $J \ll \kappa_s$ in our device. The qubit state is verified by the converted optical Stokes output field from the electro-optic device,

$$\hat{a}_{o,\mathrm{out}}(t) = -\sqrt{\kappa_{o,\mathrm{ex}}}\hat{a}_o(t). \tag{8}$$

D. Full optical readout model

The full optical readout of the superconducting qubit is realized by sending an optical signal together with an optical pulse to the electro-optic transducer. The converted microwave signal is used for qubit readout and reflected back to the electro-optic transducer. The reflected microwave field is subsequently converted again into the optical domain. The dynamics of the electro-optic device from Eq. 4-7 is now related to the cQED system by

$$\begin{aligned}
\hat{a}_{e,\mathrm{in}}(t) &= \eta_{e,c}\hat{a}_{c,\mathrm{out}}(t), && (9)\\
\hat{a}_{c,\mathrm{in}}(t) &= \eta_{c,e}\hat{a}_{e,\mathrm{out}}(t). && (10)
\end{aligned}$$

The superconducting qubit state thus can be verified from the reflected optical anti-Stokes field,

$$\hat{a}_{o,\mathrm{out}}(t) = \hat{a}_{o,\mathrm{in}}(t) - \sqrt{\kappa_{o,\mathrm{ex}}}\hat{a}_o(t). \tag{11}$$

E. Skewed excited state distribution

The histograms for the excited state measurements in the Jaynes-Cummings readout show an asymmetric distribution which is particularly visible for the well-separated peaks in the all-microwave readout (Supplementary Fig. 2a). In the dispersive low-power readout, such an asymmetry arises typically from decays during the readout due to a limited longitudinal coherence time T_1. The high power Jaynes-Cummings readout, however, is latching and remains in its states until the readout tone is switched off. Thus, the response should not suffer from the exponential qubit decay. Supplementary Fig. 2b shows the averaged time traces for three distinct regions in the histograms shaded by the corresponding color in panel a: 1) single-shot readout traces of a qubit prepared in the ground state that yielded a value within the FWHM of the corresponding peak (purple), 2) Values that fall within the FWHM of the excited state histogram peak after the qubit was prepared in the excited state (light purple), and 3) single-shot traces that form the asymmetric tail of the excited state histogram. Interestingly, the asymmetric tail in the excited state histogram arises from a deviation at the beginning of the readout pulse rather than the end. The averaged response for these cases (excited qubit state prepared) approaches first the ground-state readout response and switches then later irreversibly to the excited state readout signal. Further investigations are necessary to gain more insights in this behavior. While the frequency spectrum has been qualitatively predicted by theoretical models [2, 3], the temporal dynamics of the Jaynes-Cummings readout are still theoretically and experimentally rather unexplored.

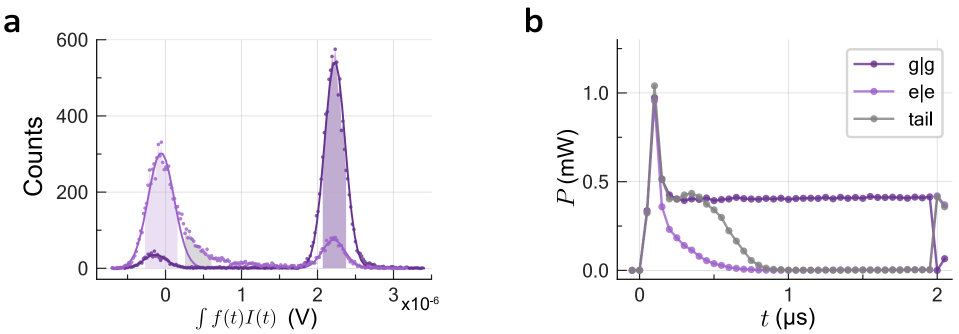

Supplementary Fig. 2. **Skewed excited state distribution.** **a**, The histograms of a Jaynes-Cummings microwave readout from Fig. 2g in the main text. Shaded regions mark selections for prepared and measured excited states (light purple), excited and measured ground states (purple) and the region (gray) where it significantly deviates from double-Gaussian fits (lines). **b**, Averaged coherent power for the respective selection highlighted in panel a. The skewness of the excited state population histogram arises from 'double' or 'delayed' switching of the cavity reflection which initially follows the ground state response before switching to the excited state cavity reflection.

II. FABRICATION

A. The cQED system

For the fabrication of the transmon qubit, a 10 x 10 mm^2 high resistivity silicon chip was cleaned using an O_2 plasma asher followed by a buffered oxide etch (BOE) dip for 30s. The chip is further cleaned by sonicating in 50 °C acetone for 10 mins and finally rinsed with isopropyl alcohol (IPA). The cleaned chip is covered with double layer of MMA/PMMA resist to pattern the qubit with two capacitor pads of area 330 x 550 μm^2 separated by a 400 μm distance along with a Josephson junction in the center of the gap between the two capacitors. The entire process is performed in a single e-beam lithography (EBL) step using the Dolan bridge process for an Al/AlO$_x$/Al junction of area 200 x 300 nm^2. For the metal deposition, an in-situ gentle argon ion milling is performed to clean any resist residues from the surface of the silicon. The Josephson junction is then fabricated by evaporating 60 nm aluminum followed by static oxidation in pure O_2 environment and then a 120 nm thickness aluminum layer is evaporated. The residual metal is lifted off using dimethyl sulfoxide (DMSO) at 80 °C for 3 hours followed by an acetone and IPA rinse. Finally, the sample is covered with S1805 photo resist and UV tape as the 10 x 10 mm^2 chip is diced into three 10 x 2.5 mm^2 pieces with one qubit on each diced chip.

The transmon qubit is strongly coupled to a 23x15 mm rectangular waveguide cavity with rounded sidewalls made of 6061 Aluminum alloy. Indium seals the seam between the top and bottom part of the cavity. The fundamental mode can be tuned by a 8x8 mm Aluminum plate attached to an Aluminum rod inserted from an opening in the cavity housing. The central position of the tuner at the strongest field strength allows a large tuning range from $\approx$ 9 GHz to $\approx$ 7 GHz by the 5 mm range of motion of an Attocube ANPz101 piezomechanical nanopositioner.

The cQED system is mounted on a gold-plated oxygen-free high-conductivity copper holder and surrounded by a μ-metal shield which protects it from static magnetic fields. Additionally, the magnetic shield of the lowest temperature stage of our Bluefors LD250 dilution refrigerator [5] is painted by an infrared absorptive material.

B. The electro-optic transceiver

The electro-optic device comprises an optical cavity by means of a $LiNbO_3$ whispering gallery mode (WGM) resonator and a cylindrical 3D cavity made of pure Aluminum. The center of the cavity exhibits circular protrusions from the cavity top and bottom forming a narrow, ring-shaped gap. As the rings clamp the optical resonator close the optical modes confined at the rim and additionally strongly confine the electric field of the $m = 1$ microwave resonance, the overlap between both fields is optimized. The microwave resonance can be tuned by roughly 500 MHz inserting an aluminum cylinder into the cavity by another Attocube piezomechanical nanopositioner. GRIN lenses focus the optical input and output from the optical fiber on a diamond prism coupled to the WGM resonator. More details about fabrication and characterization can be found in [6].

The device is identical to previous works [6–8] but suffered from an internal linewidth increase from $2\pi \times 7$ MHz to $\approx 2\pi \times 40$ MHz. Together with with a reduced mode matching factor for the coupling between the single mode optical

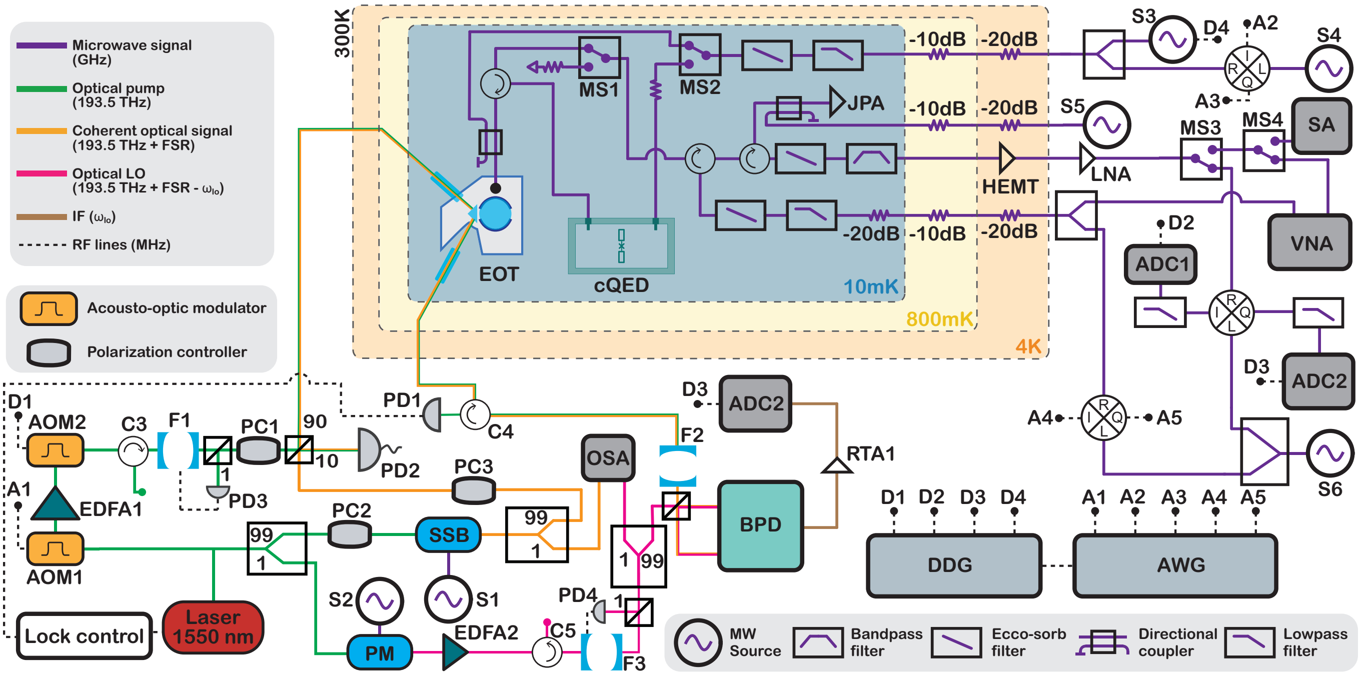

Supplementary Fig. 3. **Experimental setup**. **Optical setup (bottom left)** A telecom laser (Toptica DLC CTL 1500) gets split into the optical pump (green) and the optical signal (orange) and optical local oscillator (LO) for heterodyne detection (pink) respectively. The optical pump is amplified by an EDFA (Amonics AEDFA-PM-NS-200-10-23-M-FA) and pulse-shaped by two acousto-optical modulators (AOM, Fibre-Q T-M200-0.1C2J-3-F2P) with corresponding input signal from an arbitrary waveform generator (AWG, port A1, Spectrum Instrumentation M4i.6622-x8) and a digital delay/pulse generator (DDG, Stanford Research Systems DG645). An in-house filter cavity (F1) with analog PI-lock (PD3) removes the broadband noise from the EDFA amplification. The optical signal (orange) is generated by a single side-band modulator (SSB, Thorlabs DQPSK optical modulator) at the frequency of the Anti-Stokes mode, i.e. blue-shifted by 8.806 GHz. The optical LO (pink) is detuned from the laser frequency by a phase modulator (PM) and amplified. The PM's spurious modes are consequently cleaned by a tunable filter cavity (F3, MicronOptics FFPI) locked by software controlling a Peltier element. The optical output signal is cleaned from the reflected pump by another tunable filter (F2, MicronOptics FFPI) and finally combined with the optical LO for heterodyne detection on a balanced photodetector (Thorlabs PDB470). The downconverted signal is sent to an analog-digital converter (ADC2, port 2, AlazarTech ATS9870). **Microwave setup (top right)** An AWG (Quantum Machines OPX+) generates the qubit drive pulse via an IQ-mixer (ports A2, A3, Marki IQ-4509MXP). The pulse is sent either to the weakly coupled port of the qubit-cavity system (cQED system) or to the electro-optic transducer (EOT) for direct conversion measurements. The Quantum machines OPX+ also generates the qubit readout signal via an IQ-mixer (ports A4, A5, Marki IQ-0618MXP). The readout pulse is sent to the strongly coupled port of the cQED system. After being reflected there and at the EOT's microwave cavity, the pulse is routed via a reflective Josephson parametric amplifier [9], a cryogenic HEMT amplifier(Low Noise Factory LNF-LNC6_20C), and a room temperature low-noise amplifier (Agile AMT-A0067) to an IQ mixer. The downconverted signal from the IF port is sent to ADC2 (port 1) and the digitizer of Quantum Machines OPX+ (ADC1). Timing and synchronisation is controlled by the DDG. A vector network analyzer (VNA, Rohde and Schwartz ZVL13) can also be used to characterize the microwave setup. The microwave output or the signal from a 50 Ω termination (microwave switch MS1) may be sent to a spectrum analyzer (Rohde and Schwarz FSW26) for noise measurements. Further Acronyms: PC - polarization controller, PD - photodetector(Thorlbabs PDA50B2, PDA05CF2, PDA20CS2)

fiber to the electro-optic device, this results in an almost two orders of magnitude decrease in conversion efficiency. We attribute the linewidth broadening to damages in the $LiNbO_3$ disc induced by the clamping rings due to thermal expansion during warmup. A soft Indium clamping in the center of the disc far from the optical modes resolved this issue in a recent device and the optical linewidth remains constant during cooldown and warmup.

III. EXPERIMENT

The experimental setup is described in Supplementary Fig. 3 and the device parameters are listed in Supplementary Tab. 2.

Supplementary Tab. 2. **Device parameters.**

Parameter	Symbol	Value
Qubit frequency	ω_q	$\omega_\mathrm{q}/2\pi = 6.251\mathrm{GHz}$
Qubit-cavity coupling	g_qc	$g_\mathrm{qc}/2\pi = 326\mathrm{MHz}$
Qubit anharmonicity	ν	$\nu/2\pi = 201\mathrm{MHz}$
Dispersive shift	χ	$\chi/2\pi = 6.6\mathrm{MHz}$
Cavity frequency	ω_c	$\omega_\mathrm{c}/2\pi = 8.806\mathrm{GHz}$
Cavity linewidth	κ_c	$\kappa_\mathrm{c}/2\pi = 1.4\mathrm{MHz}$
Weak port coupling	$\kappa_\mathrm{c,w}$	$\kappa_\mathrm{c,w}/2\pi = 100\mathrm{kHz}$
Cavity internal loss	$\kappa_\mathrm{c,\ int}$	$\kappa_\mathrm{c,\ int}\ /2\pi = 300\mathrm{kHz}$
Qubit lifetime	T_1	$T_1 = 40\mu\mathrm{s}$
Ramsey time	T_2	$T_2 = 1.5\mu\mathrm{s}$
Optical cavity frequency	ω_o	$\omega_\mathrm{o}/2\pi = 193.4\mathrm{THz}$
Optical cavity external coupling	$\kappa_\mathrm{o,ext}$	$\kappa_\mathrm{o,\ ext}\ /2\pi = 44\mathrm{MHz}$
Optical cavity linewidth	κ_o	$\kappa_\mathrm{o}/2\pi = 81\mathrm{MHz}$
EO Microwave cavity frequency	ω_e	$\omega_\mathrm{e}/2\pi = 8.806\mathrm{GHz}$
EO Microwave cavity external coupling rate	$\kappa_\mathrm{e,ext}$	$\kappa_\mathrm{e,ext}/2\pi = 3.42\mathrm{MHz}$
EO Microwave cavity linewidth	κ_e	$\kappa_\mathrm{e}/2\pi = 9.69\mathrm{MHz}$
Vacuum electro-optical coupling	g_eo	$g_\mathrm{eo}/2\pi = 30$ Hz

[1] M. D. Reed, L. DiCarlo, B. R. Johnson, L. Sun, D. I. Schuster, L. Frunzio, and R. J. Schoelkopf, Phys. Rev. Lett. **105**, 173601 (2010).
[2] M. Boissonneault, J. M. Gambetta, and A. Blais, Physical Review Letters **105**, 100504 (2010).
[3] L. S. Bishop, E. Ginossar, and S. M. Girvin, Physical Review Letters **105**, 100505 (2010).
[4] C. W. Gardiner, Physical Review Letters **70**, 2269 (1993).
[5] Bluefors LD Dilution Refrigerator Measurement System, https://bluefors.com/products/dilution-refrigerator-measurement-systems/ld-dilution-refrigerator-measurement-system/, accessed 23-10-2023.
[6] W. Hease, A. Rueda, R. Sahu, M. Wulf, G. Arnold, H. G. Schwefel, and J. M. Fink, PRX Quantum **1**, 020315 (2020).
[7] R. Sahu, W. Hease, A. Rueda, G. Arnold, L. Qiu, and J. M. Fink, Nature Communications **13**, 1276 (2022).
[8] R. Sahu, L. Qiu, W. Hease, G. Arnold, Y. Minoguchi, P. Rabl, and J. M. Fink, Science **380**, 718 (2023).
[9] P. Winkel, I. Takmakov, D. Rieger, L. Planat, W. Hasch-Guichard, L. Grünhaupt, N. Maleeva, F. Foroughi, F. Henriques, K. Borisov, J. Ferrero, A. V. Ustinov, W. Wernsdorfer, N. Roch, and I. M. Pop, Physical Review Applied **13**, 24015 (2020).
